# Characterization of the N-Terminal Domain of BteA: A *Bordetella* Type III Secreted Cytotoxic Effector

**DOI:** 10.1371/journal.pone.0055650

**Published:** 2013-01-30

**Authors:** Chen Guttman, Geula Davidov, Hadassa Shaked, Sofiya Kolusheva, Ronit Bitton, Atish Ganguly, Jeff F. Miller, Jordan H. Chill, Raz Zarivach

**Affiliations:** 1 Departments of Life Sciences, Ben-Gurion University of the Negev, Be'er Sheva, Israel; 2 National Institute for Biotechnology in the Negev (NIBN), Ben-Gurion University of the Negev, Be'er Sheva, Israel; 3 Ilse Katz Institute for Nanoscale Science and Technology, Ben Gurion University of the Negev, Beer Sheva, Israel; 4 Department of Chemical Engineering, Ben-Gurion University of the Negev, Beer-Sheva, Israel; 5 Department of Chemistry, Bar Ilan University, Ramat Gan, Israel; 6 Department of Microbiology, Immunology and Molecular Genetics, David Geffen School of Medicine, University of California Los Angeles, Los Angeles, California, United States of America; Pasteur Institute, France

## Abstract

BteA, a 69-kDa cytotoxic protein, is a type III secretion system (T3SS) effector in the classical *Bordetella*, the etiological agents of pertussis and related mammalian respiratory diseases. Currently there is limited information regarding the structure of BteA or its subdomains, and no insight as to the identity of its eukaryotic partners(s) and their modes of interaction with BteA. The mechanisms that lead to BteA dependent cell death also remain elusive. The N-terminal domain of BteA is multifunctional, acting as a docking platform for its cognate chaperone (BtcA) in the bacterium, and targeting the protein to lipid raft microdomains within the eukaryotic host cell. In this study we describe the biochemical and biophysical characteristics of this domain (BteA287) and determine its architecture. We characterize BteA287 as being a soluble and highly stable domain which is rich in alpha helical content. Nuclear magnetic resonance (NMR) experiments combined with size exclusion and analytical ultracentrifugation measurements confirm these observations and reveal BteA287 to be monomeric in nature with a tendency to oligomerize at concentrations above 200 µM. Furthermore, diffusion-NMR demonstrated that the first 31 residues of BteA287 are responsible for the apparent aggregation behavior of BteA287. Light scattering analyses and small angle X-ray scattering experiments reveal a prolate ellipsoidal bi-pyramidal dumb-bell shape. Thus, our biophysical characterization is a first step towards structure determination of the BteA N-terminal domain.

## Introduction

*Bordetella pertussis* is the causative agent of pertussis (also known as "whooping cough"), a highly contagious disease which remains one of the world' leading causes of vaccine-preventable deaths. Approximately 100,000 cases were reported in 2010 while 200,000 deaths were estimated in 2008 alone; case-fatality rates in developing countries are estimated to be as high as 4% in infants [1]. Among the ‘classical’ *bordetella* (comprising *B. pertussis*, *B. parapertussis* and *B. bronchiseptica*), *B. bronchiseptica* is known to use the type III secretion system (T3SS) to establish persistent colonization of the trachea and to modulate the host immune response [2]. T3SS is a multi-component secretion machinery that secretes effectors directly into the cytosol of the host cell with the aid of a designated chaperone [3]. To date, BteA is the only confirmed T3SS effector identified in *Bordetella bronchiseptica*, and it is highly conserved (protein identities greater than 95%) among the three sub-species.

BteA is a 69-kDa protein that, upon translocation, induces rapid non-apoptotic eukaryotic cell death via an unknown mechanism [4], [5]. *Bordetella* strains bearing null mutations of the *bteA* gene were shown to have negligible cytotoxic levels similar to type III deficient mutants indicating that this molecule is absolutely required for T3SS mediated cytotoxicity and indicating a significant role for BteA in T3SS function during *Bordetella* infection [5]. BteA is also indirectly involved in the dephosphorylation of tyrosine-phosphorylated proteins in the host [6]. In addition, Han et al [7] have shown that BteA expression is upregulated in certain clinical strains of *B. pertussis* but not in strains used for preparing vaccines. Recently, Ahuja et al have shown that hypercytotoxicity and hypervirulence capabilities of virulent human-associated complex IV *B. Bronchiseptica* strains were dependent on BteA loci and its expression [8].

It has previously been shown that the C-terminal domain is required for the cytotoxicity of BteA, while a portion of the N-terminal domain (1–130) binds the putative chaperone, BtcA [4]. The N-terminal is also responsible for BteA localization at Ezrin-rich lipid rafts in mammalian cells and residues 34–112 are homologous to the lipid raft targeting (LRT) domain of RTX toxins. The structural properties of the N-terminal domain as well as the mechanisms by which it targets BteA to lipid rafts, remain unknown [4]. A step towards a structural understanding of the BteA N-terminal domain was made in a previously deposited NMR structure of a fragment corresponding to residues 115–220 derived from the *B. parapertussis* homolog (PDB code 2JPF, unpublished). This structure, determined by the Structural Genomics consortium at Toronto, showed residues 115–145 to be unstructured, and found considerable helical content for residues 145–220. Although it affords limited information regarding secondary structure composition, it does not address most of the N-terminal region, and thus fails to meet the need for a comprehensive structural investigation of this domain.

In the current work we present an extensive biochemical and biophysical analysis of the recombinant N-terminal domain of BteA, extending the domain to the first 287 amino acids (BteA287). We demonstrate that unlike the full length protein, BteA287 is soluble and monomeric in nature, with a tendency to aggregate at elevated concentrations. Using circular dichroism, light-scattering techniques, nuclear magnetic resonance (NMR) and small angle X-ray scattering (SAXS) experiments we further show that BteA287 has rich alpha-helical content and adopts the form of a prolate ellipsoid bi-pyramidial dumb-bell. Thus we lay the foundations for the determination of the complete structure of the BteA N-terminal domain.

## Materials and Methods

### Cloning, Expression and purification of BteA and BteA287

The BteA gene fragments 1–1974 (corresponding to residues 1–658), 1–861 bp (corresponding to residues 1–287) and gene fragment 94–861 bp (corresponding to residues 32–287) were amplified from B. Bronchiseptica genomic DNA using the Polymerase Chain Reaction (PCR) with forward primer CAGATCCATATGTTGAGCAACAACGTCAATCCG (full length BteA) or with GGCCTGGTGCCGCGCGGCAGCCATATGTTGAGCAACAACGTCAATCCG (fragment 1–287) or with forward primer GGCCTGGTGCCGCGCGGCAGCCATATGCGCCTGCTCGAGCCGAACAAC (fragment 32–287) and reverse primers CAGATCAGATCTGTTTAAACTCAATGGTGATGGTGATGGTGGCCCTGGAAGTACAGGTTTTCGCGTGCGCGTAGATTCAGCGCCG (full length BteA) or withGTGCTCGAGTGCGGCCGCAAGCTTTCATCAGTCGTCGGCCTGCTGCAGGGC (BteA287) containing the NdeI, BglII, PmeI and HindIII restriction sites (marked with underline). The established full length BteA amplicon was cloned into pET11a vector (Thermo Scientific, Asheville, NC) through NdeI and BamHI sites while BteA287 were cloned through the respective restriction sites within pET28a(+) vector (Thermo Scientific, Asheville, NC). The ligated plasmids were transformed into BL21 (DE3) competent bacteria cells after which selected colonies were grown to mid-exponential phase. At this point expression of the proteins was induced by the auto induction protocol [9] or by addition of isopropyl β-D-1-thiogalactopyranoside (IPTG) to 1 mM final concentration for 18 hr at 20 °C. For preparation of isotopically labeled NMR samples cells were grown in M9-based minimal medium containing 1 g/L ^15^NH_4_Cl (for the ^15^N-labeled sample) or D_2_O-based M9 medium containing 1 g/L ^15^NH_4_Cl, 2.5 g/L ^13^C_6_-glucose and 1 g/L DCN-Isogro (Sigma-Aldrich, Rehovot, Israel) hydrolysate (for the ^2^H,^13^C,^15^N-labeled sample)[10]. Cells were collected by centrifugation at 6000 rpm for 7 min at 4 °C after which the pellet was resuspended with binding buffer (20 mM Imidazole, 300 mM NaCl, 20 mM Tris pH 8, 0.02% Triton X-100). The cells were lysed with French Press (Thermo Scientific, Asheville, NC) and centrifuged at 45,000 rpm for 45 min at 4 °C. Protein batch binding assay was conducted by applying the supernatant to buffer equilibrated Ni-NTA beads (Novagen), followed by 3 washing steps using Econo-Column (Bio-Rad, Hercules, CA): buffer 1 (25 ml of 300 mM NaCl, 20 mM Tris pH 8, 20 mM imidazole), buffer 2 (50 ml of 600 mM NaCl, 20 mM Tris, pH 8, 30 mM imidazole) and buffer 3 (25 ml of 300 mM NaCl, 20 mM Tris pH 8, 40 mM imidazole). Elution was performed in the presence of elution buffer (50 mM NaCl, 20 mM TRIS pH 8, 300 mM imidazole) after which eluted samples of BteA287 and BteA32–287 were equilibrated overnight against dialysis buffer (50 mM NaCl, 20 mM HEPES pH 7.5) at 4 °C with 7000 MW cutoff SnakeSkin™ (Thermo Scientific, Asheville, NC). Equilibrated samples were purified with Mono-Q anion exchange column (GE healthcare, Little Chalfont, UK) utilizing linear gradient with elution buffer (2 M NaCl, 20 mM HEPES pH 7.5). Selected fractions were pooled, fixed to protein's buffer (150 mM NaCl, 20 mM HEPES pH 7.5) and concentrated to 20 mg/ml (588 µM).

### MALDI-TOF/MS analysis

Matrix was prepared by dissolving sinapinic acid (Sigma-Aldrich, Rehovot, Israel) in TA (33% Acetonitrile, 0.1% TFA) until saturation occurred. The protein sample was mixed with the matrix at 10:1 and 100:1 v/v (matrix: sample) ratios. The mixture (1 µl) was dispensed on the MALDI target plate and dried at ambient temperature. Samples were analyzed on a Reflex IV (BrukerDaltonics, Bremen, Germany) MALDI-TOF mass spectrometer using 337 nm radiation from a nitrogen laser. The spectra were recorded in linear mode within a mass range from m/z 20,000 to 150,000.

### Limited proteolysis

Trypsin (Sigma-Aldrich, Rehovot, Israel), dissolved at 1.5 mg/ml (64 µM) in 1 mM HCl and 2 mM CaCl_2_, was added to purified BteA samples at a ratio of 1:130 and the reaction was incubated on ice for 5, 10, 20, 30 and 60 minutes. At the indicated time points the reaction was quenched with the addition of sample buffer and the samples (including a non-digested sample) were separated on a 10% SDS-Polyacrylamide gel.

### Analytical size exclusion chromatography and molecular weight determination

Purified protein (20 mg/ml (588 µM)) was loaded onto a Suprdex 75 10/300 (GE healthcare, Little Chalfont, UK) equilibrated with 20 mM Tris buffer pH 8, 100 mM NaCl and elution volume was monitored via absorbance at 280 nm. A calibration curve was generated by plotting the elution volume of a protein standard kit (GE healthcare, Little Chalfont, UK) against their known molecular weight. The elution volume of BteA287 was used to extract the molecular weight from the established curve.

### Circular dichroism analysis

Circular dichroism measurements were conducted with a J750 Spectropolarimeter (Jasco Inc, Mary's Court, Easton, USA) equipped with a Pelletier device. BteA287 and BteA32-287 protein samples were prediluted to 6 µM in buffer containing 50 mM NaCl, 20 mM Tris pH 8 and measured with a 0.1 cm optical path Suprasil quartz cuevette (Hellma GMBH & Co., Müllheim, Germany). Spectra profiles of the samples were measured at a wavelength range of 190–240 nm at ambient temperature with bandwidth set to 1 nm, scan speed set to 10 nm⋅min^−1^ and a time constant of 4 seconds. Thermal denaturation experiment of BteA287 was conducted by monitoring the dichroic absorption at wavelength of 222 nm as a function of increased temperature varying from 25 °C to 95 °C at a heating rate of 1.0 °C⋅min^−1^. The thermodynamic parameters associated with the temperature-induced denaturation were obtained by nonlinear, least-squares analysis of the temperature dependence of CD, and a two-state denaturation process was assumed during curve-fitting analysis.

### Analytical ultracentrifugation

Ultracentrifugation experiments were performed using an XL-I analytical ultracentrifuge, equipped with An-60Ti rotor and absorbance optics (Beckman-Coulter Inc., Brea, CA). Sedimentation equilibrium data were collected at 20 °C and 15,000 rpm in double-sector cells of 12 mm thickness, adapted to the absorption at 280 nm. The experiment was carried out in 100 mM NaCl, 20 mM Tris buffer pH 8 at three different concentrations (1.0, 0.75, and 0.5 mg/ml (30 µM, 22 µM and 15 µM)). Sedimentation curves thus obtained were analyzed with in-house MATLAB-based scripts using a non-linear least-squares approach to extract molecular weight information. Models tested were (i) monomer, (ii) monomer-dimer equilibrium, (iii) monomer-oligomer equilibrium, (iv) monomer with high MW aggregate, and (v) monomer-dimer equilibrium with high MW aggregate. Model selection was based on a F-statistic obtained from comparison of residuals.

### Dynamic light scattering and BteA287 aspect ratio determination

A CGS-3 goniometric Dynamic Light Scattering System (ALV-GmbH, Langen, Germany) was used for particle distribution and size determination of 2 mg/ml (58 µM) of BteA287 in 20 mM Tris buffer pH 8 and 100 mM NaCl. For the determination of BteA287 aspect ratio, it was assumed to fit into a prolate ellipsoid with short and long axes of a and b, respectively. The molecular volume formula of BteA287 was then represented as 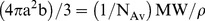
(1)where MW is BteA287's molecular weight, N_Av_ is Avogrado's number, and ρ is the protein specific density, calculated from amino acid sequence as 1.38 [11]. The long axis b was determined from DLS (35⋅10^−8^ cm), allowing the estimation of short axis a and aspect ratio b/a.

### Multiangle light scattering

Purified protein (15 mg/ml (441 µM)) was loaded onto sequentially-coupled 10 µM-particle size SUPREMA 100A and 1000A columns (PSS, Mainz, Germany), equilibrated with buffer (20 mM Tris pH 8.0 and 100 mM NaCl), and connected in line with DAWN multiangle light-scattering equipment coupled to an interferometric refractometer (Wyatt Technologies, Santa Barbara, CA). Data analysis was done in real time using ASTRA (Wyatt Technologies, Santa Barbara, CA) and molecular masses were calculated using the Debye fit method.

### NMR data acquisition

For acquisition of NMR data BteA287 samples were prepared in 20 mM NaH_2_PO_4_/Na_2_HPO_4_ buffer (pH 7.3), 100 mM NaCl and 7% D_2_O. Typical protein concentrations were 80–600 µM. Data were acquired on a DRX 700 MHz spectrometer equipped with z-gradients and a cryoprobe at 303 K. HSQC and TROSY-HSQC (tr-HSQC) spectra were acquired with 1024 (128) complex points in the ^1^H (^15^N) dimension and 4–8 transients per hypercomplex point, with echo-antiecho mode used for quadrature detection. Typically, 20–40 min were required per experiment. Diffusion measurements employed a bipolar pulse longitudinal eddy-current delay (BPP-LED)-based experiment [12] acquired at 298 K with a diffusion time of Δ = 300 ms, total gradient duration of τ = 4.8 ms, an eddy-delay of τ_e_ = 5 ms, and relative z-gradient strengths of 5–50% corresponding to gradients of 2.57–25.7 gauss/cm. The diffusion coefficient was obtained by fitting the decay of intensity observed in the methyl region of the spectra acquired for 8–10 different gradient strengths to the equation
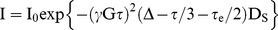
(2)where γ is the proton gyromagnetic ratio (2.67×10^8^ T^−1^s^−1^), G is the applied magnetic gradient field and *τ* is the total length of the bipolar gradient, *D*_s_ is the diffusion coefficient, and all other variables defined as above [13]. Typical errors in the determination of *D*_s_ were 1–2%. Gradient strengths were calibrated using a sample of 8 mg/ml (0.57 mM) hen egg-white lysozyme (Sigma) in 93:7 H_2_O:D_2_O with a known value at 298 K of *D*_s_ = 11.1×10^−11^ m^2^/sec [14], [15].

### SAXS measurements

Synchrotron radiation X-ray scattering data were collected at the X33 beam line of the EMBL, Hamburg Outstation (DORIS III storage ring at DESY) [16] equipped with an automatic sample changer [17]. Glass capillaries were filled with solutions of purified BteA287 (1, 2 and 10 mg/ml concentrations (30 µM, 60 µM and 300 µM)) with sample temperature set to 10 °C). Using wavelength λ = 1.56 Å, data was collected using a MAR345 image plate detector and sample detector distance of 2.7 m and covering the momentum transfer range 0.08<s<0.45 nm-1 (s = 4π sin(θ)/λ where 2θ is the scattering angle) and 3-min exposure times. Radiation damage was monitored using standard procedures. 2D SAXS images were azimuthally averaged to produce 1D intensity profiles using FIT2D. For background subtraction, scattering profiles were obtained for capillaries filled with solvent.

### SAXS data analysis and envelope model

The radius of gyration (Rg) was evaluated using the Guinier approximation [18]. The GNOM program was used to obtain the Pair-distance distribution functions, the corresponding maximum dimension of protein complexes (Dmax) and to determine the value for Rg from the entire scattering profile [19]. *Ab initio* envelopes were generated by the program DAMMIN (Svergun, 1999) using atomic radii set to the dummy atom packing radius determined by DAMMIN without imposing symmetry operation [19]. The generated envelope models (DBMs) were fitted on the core structure of the deposited solution NMR (2JPF, residues Gln30-Arg99) using the Coot software [20] and visualized by PyMOL [21]. Defining the core residues of the 2JPF ensemble was conducted using OLEDARDO [22].

## Results

### BteA is predicted to contain two domains and a T3SS-secretion signal peptide

**Figure 1:**
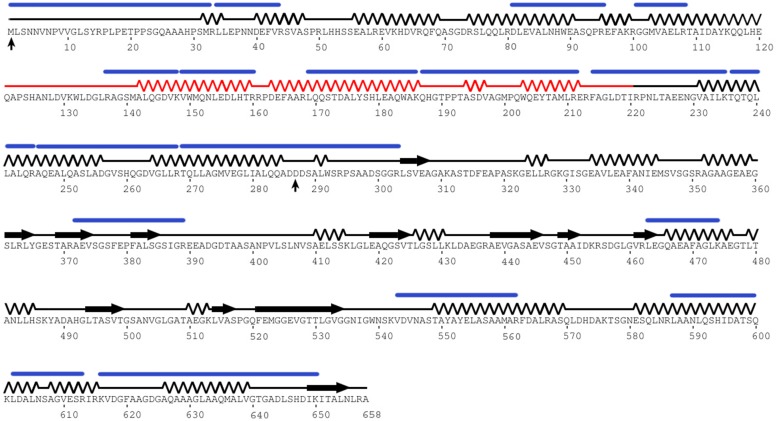
Secondary structure prediction of BteA superposed on established experimental data. The sequence of BteA was subjected to secondary structure meta-server analysis which was plotted as line for coil, spring for alpha-helix and arrow for beta sheet. Vertical arrows mark the boundaries of BteA287 fragment. Experimentally-derived secondary structure topology (PDB code 2JPF, aa 121–220) is shown in red. BteA-derived peptides from peptide fingerprinting MS/MS experiment (see Materials and Methods) were plotted as blue lines.

Previous publications have indicated that BteA exhibits multi-domain functionality [4], [5], thus we were interested in performing sequence-based bioinformatics analysis of BteA to determine the boundaries of these domains. Employing the ProteinCCD meta-server [23], PSIPRED [24] and the PDBsum SAS server [25] we compiled a secondary structure profile for BteA (Figure 1). BteA is predicted to be composed of an unstructured first 31 amino acids followed by ∼260 residues which are predicted to fold as alpha-helices (domain #1, hereafter "N-terminal"). The N-terminal is followed by a mixture of well spaced loops, short beta-sheets and alpha-helices up to residue 409 (hereafter "linker"). From the C-terminal domain, from the center domain up to the protein terminus is predicted to fold as interchanging alpha-helices and beta-sheets (domain #2, hereafter "C-terminal"). This is in agreement with the previously deposited NMR structure of residues 115-220 from B. parapertussis (PDB code 2JPF, unpublished).

It was previously shown that T3SS effectors are characterized by an unstructured signal peptide formed by their first 30 to 50 residues [26], [27] which are, among other characteristics, enriched with polar residues and lack acidic residues in the first 12 amino acids [28], [29]. We manually analyzed the first 31 residues of BteA to characterize its putative secretion signal and determined that it fulfils five of the six-predictive criteria suggested by Petnicki-Ocwieja et al [29]. In addition we found residues 1–31 to be mostly composed of aliphatic residues, which might suggest possible hydrophobic interactions with BteA's cognate chaperon or with host target protein(s). The BteA sequence was also analyzed by the SIEVE server which predicts whether a protein is a potential T3SS effector[26]. A Z-score of 2.65 (raw discriminate –0.56) classified BteA as a T3SS effector in agreement with previous publications [4], [5] as well as the prediction of the N-terminal unstructured region. Thus we can conclude that BteA multi-functionality, previously only empirically determined, is supported by secondary structure and secretion signal predictions.

### BteA is composed of a stable N-terminal domain

**Figure 2:**
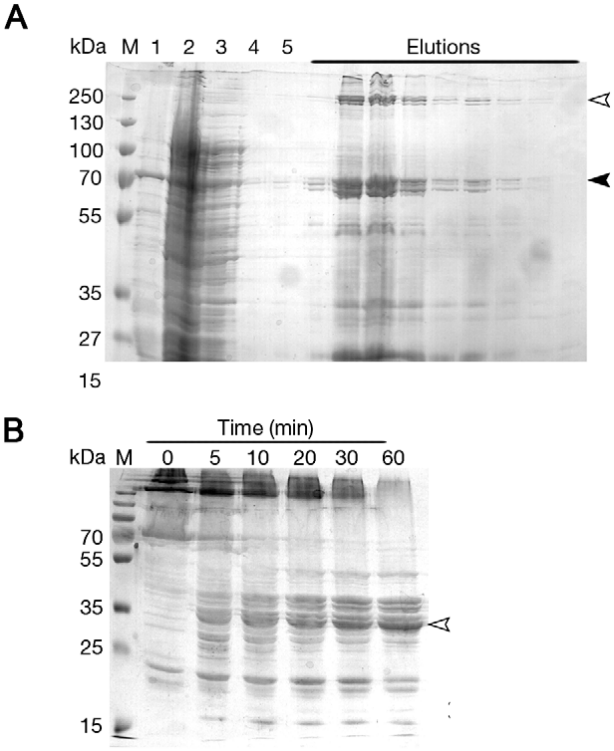
SDS-PAGE analysis of BteA purification and limited proteolysis experiments. (**A**) Ni^2+^-affinity chromatography purification of BteA showing fractions of: 1, pellet; 2, flow through; 3, wash 1; 4, wash 2; 5, wash 3; Elutions, selected elution fractions; M, marker. Black and white arrows indicate BteA monomer and oligomer respectively. (**B**) Limited proteolysis experiment was conducted on purified BteA after which the reaction was quenched by the addition of sample buffer and separated on 12.5% SDS-polyacrylamide.

For the purpose of biochemical characterization we cloned the BteA ORF into a commercial bacterial expression system, and expressed and purified the protein using Ni^2+^-affinity chromatography. The elution fraction exhibited pronounced viscosity and SDS-polyacrylamide gel electrophoresis (SDS-PAGE) analysis revealed multiple high-molecular weight bands (Figure 2, white arrow) accompanying a relatively pure BteA monomer (Figure 2a, black arrow). This evidence of aggregation or polymerization motivated us to independently express, purify and characterize each BteA domain.

**Figure 3:**
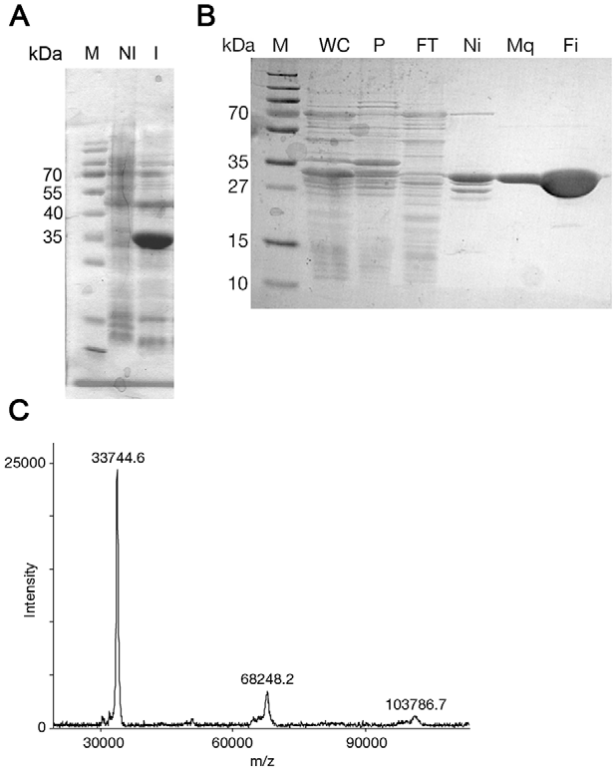
Expression and purification analysis of BteA287. (**A**) SDS-PAGE analysis of non-induced (NI) and IPTG-induced (I) samples that were taken from overnight grown cultures and were lysed by the addition of sample buffer followed by SDS-PAGE. (**B**) SDS-PAGE analysis of BteA287 Ni^2+^-affinity chromatography (AC) and anionic ion-exchange chromatography (IEC) purifications. Lanes are labeled as follows, M, marker; WC, whole cell lysate; P, pellet; FT, flow through; Ni, Elution from AC column; Mq, elution from IEC column; Fi, concentrated BteA287 sample. (**C**) MALDI/TOF-MS analysis of purified BteA287 with corresponding masses (in Daltons) indicated above main peaks.

To support our bioinformatic analysis with empirical evidence and to identify the boundaries of each domain, we conducted a limited proteolysis experiment on the purified fractions with diluted trypsin (Figure 2b). The accumulation of a major fragment migrating as a 34 kDa polypeptide (Figure 2b, white arrow) suggested this is a stable BteA domain. We excised the protein band from the gel and following trypsinization the resulting peptides were submitted to ESI-MS/MS analysis. The analysis identified 18 distinct peptide fragments, of which 12 were mapped to the first 300 residues (Fig 1, blue lines), thus recapitulating our bioinformatics analysis prediction profile. Therefore, we proceeded to express and purify this BteA N-terminal domain for biochemical and structural characterization, choosing to subclone fragment 1–287 (hereafter, "BteA287", Figure 1, black arrows) to avoid the 290–320 region predicted to be mostly unstructured. The fragment encoding BteA287 was cloned into an expression vector containing a hexa-histidine N-terminal tag and large scale purification was conducted. Expression assessment by SDS-PAGE analysis showed that this fragment migrated at the expected band size (34 kDa) and was highly expressible when induced for 18 hours either under IPTG (Figure 3A) or an autoinduction protocol. A large scale expression and purification scheme utilizing Ni^2+^ affinity column followed by anion ion-exchange chromatography has generated large quantities of BteA287 at over 95% purity (Figure 3B). Matrix-assisted laser desorption/ionization time-of-flight mass spectrometry analysis (MALDI/TOF-MS) produced a major peak which corresponds to the mass of 33.7 kDa further verifying the purity of the protein and the validity of the protein purification scheme (Figure 3C).

### BteA287 fold is rich with alpha helices and characterized by some aggregation

**Figure 4:**
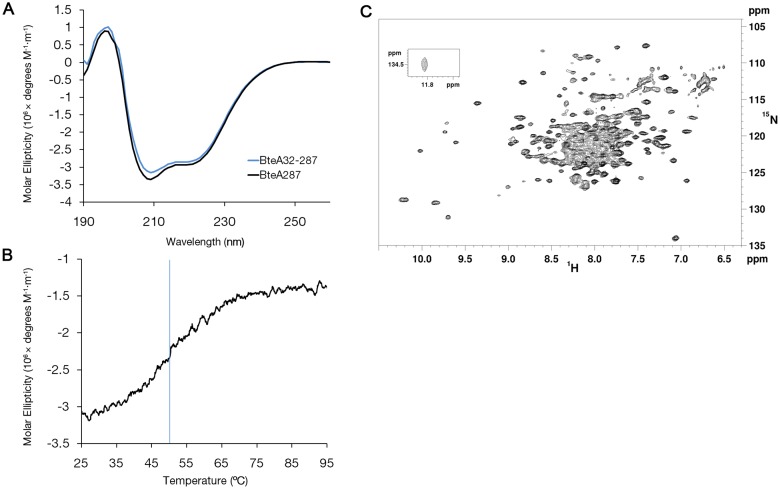
Circular dichroism analysis of BteA287 and 2D ^1^H,^15^N-TROSY-HSQC spectrum of BteA287. (**A**) Dichroic spectra for BteA287 (black line) and BteA32-287 (blue line). **(B)** Melting curve of BteA287, measured at 222 nm (blue line indicates calculated Tm). (**C**) Spectrum was acquired for a triply-labeled [^2^H,^13^C,^15^N]-BteA287 0.4 mM sample in 100 mM NaCl, 20 mM NaPi pH 7.3 at 303 K and 16.4 T. The inset shows an outlying indole NH peak.

To assess the agreement of the predicted secondary structure of BteA287 with the actual protein, we measured its circular dichroism (CD) curve at room temperature (Figure 4A, black line). The observed double minimum at 208 nm and 222 nm suggests a structure with high helical content, estimated by the K2D2 analysis algorithm [30] to be 56%, in agreement with the bioinformatic secondary structure prediction. We also expressed and purified a shorter version of BteA287 lacking the first 31 unstructured residues (hereafter "BteA32-287"), and compared its spectra to that of BteA287 (Figure 4A, blue line). We have found that BteA32-287 has very similar dichroic spectra to that of BteA287 even though it is predicted to have a higher content of alpha helices (85%) according to the K2D2 algorithm. This value corresponds with its relatively higher helical content percentage in comparison to BteA287 which further supports the assumption that the first 32 residues of BteA are unstructured. We have also analyzed the thermal stability of BteA287 via temperature dependence (25–95 °C) of the CD intensity at 222 nm exhibited a sigmoidal melting curve with an extrapolated melting temperature (Tm) of 50±2 °C (Figure 4B), further supporting our assumption that BteA287 is a stable and soluble domain. We conclude that BteA contains a stable and highly expressible N-terminal domain which is rich with alpha helical fold (residues 1–287).

We further investigated the biophysical behavior of BteA287 using nuclear magnetic resonance (NMR). For this purpose, the expression protocol was modified [10] for expression of isotopically-labeled samples with good yields, affording uniformly ^15^N-labeled (BteA^N^) and partially deuterated, uniformly [^13^C, ^15^N]-labeled (BteA^DCN^) samples. Figure 4C shows the fingerprint TROSY-^1^H,^15^N-HSQC (tr-HSQC) spectrum of BteA^DCN^ at 303 K. BteA287 appears to be a folded protein with significant helical content, as can be deduced from the highly populated central region of the spectrum and the presence of a few distinct spectral outliers. The tr-HSQC spectrum was superior in quality to an HSQC spectrum (for both BteA287 and BteA32-287), consistent with protein size. Notably, while cross-peak positions were unchanged, their intensity was not commensurate to sample concentration (as tested in the 0.1–1.0 mM range), a tendency enhanced at low salinity levels and pH values below 7.0. This suggested the presence of oligomerization or aggregation and required further investigation into the oligomeric form of BteA287.

### BteA287 oligomerization is concentration dependent

**Figure 5:**
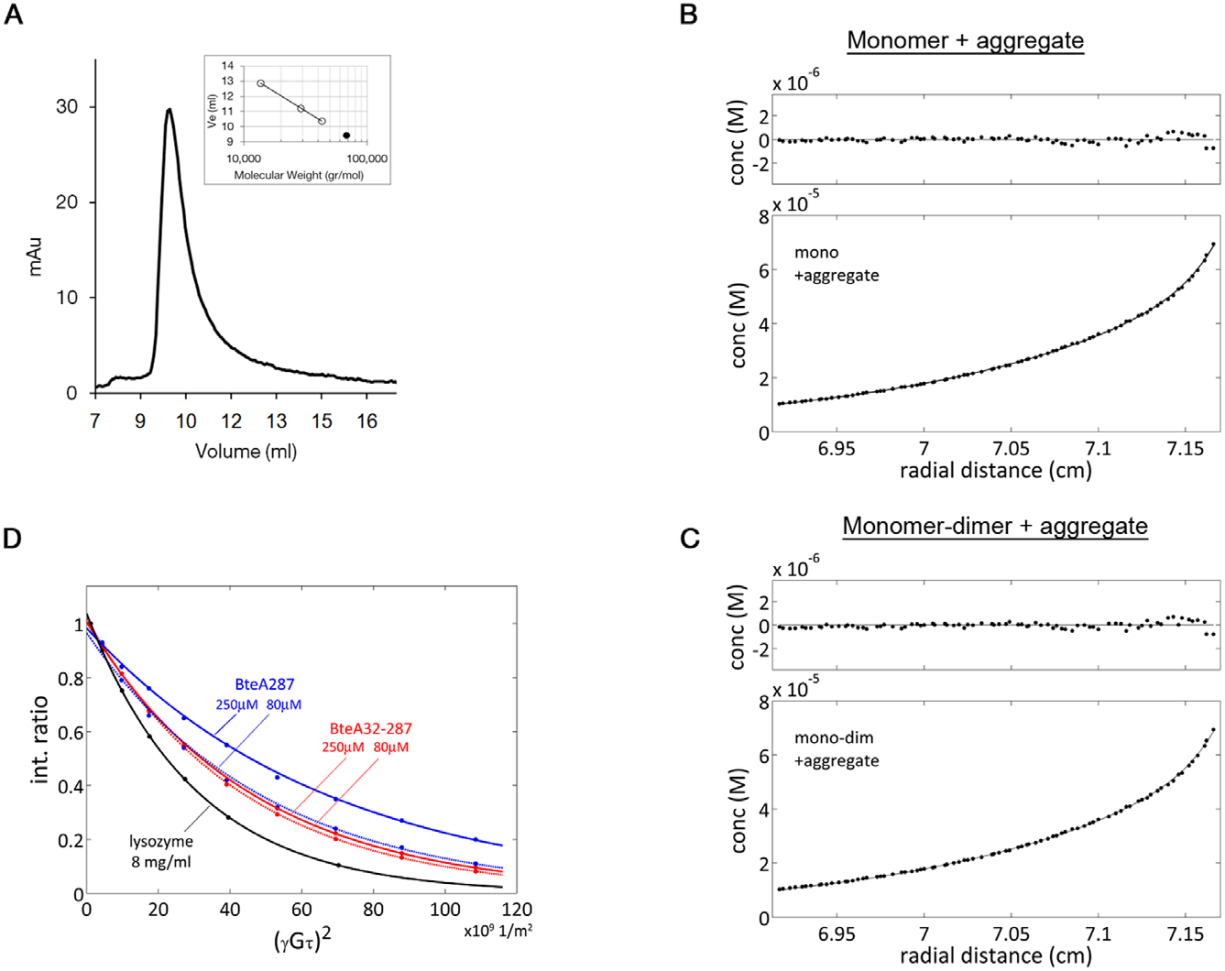
Size and oligomeric form of BteA287. (**A**) Size exclusion chromatogram of BteA287 demonstrates a single population. The elution volume of BteA287 combined with the calibration curve (inset) indicates an empirical molecular weight of 68 kDa (black point indicates the elution volume of BteA287 along the curve). (**B**, **C**) Bottom, sedimentation equilibrium curve for a 1 mg·ml^−1^ (30 µM) BteA287 sample at 15,000 rpm and 298 K in buffer containing 100 mM NaCl, 20 mM Tris pH 8. The curve was fit to a model assuming either a single monomeric species or monomer-dimer equilibrium, in both cases in the presence of a high-MW aggregate. Top, residuals of the fit. Calculated mass was 34,500 Da. (**D**) LED-BPP-based experiments were acquired to estimate the translational diffusion coefficient of BteA at 298 K. The decrease in intensity of methyl region signals is plotted against (γGτ)^2^, where γ is the proton gyromagnetic ratio (2.67·10^8^ T^−1^s^−1^), G is the applied magnetic gradient field and τ is the total length of the bipolar gradient, 4.8 ms in these experiments. Using the literature value of Ds = 11.1·10^−11^ m^2^s^−1^ for lysozyme (black) the value of G at maximal strength was determined as 0.514 Tm^−1^. Curves shown are for 250 µM BteA287 (solid blue), 80 µM BteA287 (dotted blue), 250 µM BteA32-287 (solid red), and 80 µM BteA32-287 (dotted red). In the case of BteA287, mono-exponential fits can be significantly improved using a bi-exponential fit, indicating the presence of aggregation for the longer protein.

We employed several methods in order to characterize the oligomeric nature of BteA287 over a range of concentrations. In analytical size exclusion chromatography (SEC) runs the protein eluted as a single peak at a volume corresponding to 68 kDa, as extrapolated from a calibration curve of known proteins (Figure 5A and inset), with no evidence of higher-MW species. This behavior at low concentration could be explained by dimer formation or a highly anisotropic monomer. For a view at higher concentrations we performed sedimentation equilibrium (SE) analysis at three different protein concentrations in the 15–30 µM range. The three obtained datasets were fitted to five different models (see Materials and Methods for details). Best fitting models with excellent residuals were those assuming the presence of a high-MW aggregate population (Figure 5B and 5C, lower panels). The molecular weight obtained from both models (34.5±0.5 kDa) was in excellent agreement with the size of BteA. Due to the presence of this aggregate, SE results could not establish (with statistical significance) the contribution of monomer-dimer equilibrium to BteA behavior. Since the fittings of monomer or monomer-dimer models are almost identical and observed only at high protein concentration (SE and tr-HSQC), we assume that the monomer is the prevailing form at the low to mid concentrations and that the major contributor to the shift in residuals at the high concentration part is the presence of aggregates within the sample.

To further analyze the behavior of BteA287 at higher concentrations we employed pulse field gradient NMR [12], [31] to measure the translational diffusion of BteA287 in solution, using hen egg-white lysozyme (HEWL) as a standard. BPP-LED-based experiments with a diffusion delay of 300 ms at 298 K were conducted for the 14.3 kDa HEWL and BteA287 at two concentrations, 250 µM and 80 µM (Figure 5D). HEWL diffusion under these conditions is established as *D*_s_ = 11.1×10^−11^ m^2^/sec [14], [15]. Although the expected effect of increased protein concentration on viscosity is negligible [32], the BteA287 diffusion coefficient was significantly different at varying concentrations. Mono-exponential fitting of the gradient-induced decay curve for 250 µM and 80 µM BteA287 afforded *D*_s_ values of (5.0±0.2)×10^−11^ and (6.8±0.2)×10^−11^ m^2^/sec, respectively. The former suggests for BteA a size 6–8 fold larger than HEWL, which is inconsistent with the results of the SE results. We interpreted this to mean that the ‘hard-sphere’ assumption inherent in Einstein-Stokes hydrodynamics is inapplicable to this system, and a non-negligible intermolecular interaction is affecting translational diffusion. Intermolecular interactions were also implied by the faster decay rate observed for both concentrations at low (γGτ)^2^ values compared to the overall rate, a biphasic behavior suggesting a contribution of an oligomer which dominates the observed signal at strong gradient values [33], in agreement with the sedimentation equilibrium experiment.

Since residues 1–31 are composed of mostly aliphatic residues, yet predicted as unstructured, we investigated whether they were involved in this aggregation effect. The tr-HSQC spectra of BteA 287 and BteA32-287 were very similar, supporting the notion that the first 31 residues are indeed unstructured and do not affect the overall fold of BteA287. Diffusion measurements for 250 µM and 80 µM samples of BteA32-287 yielded *D*_s_ values of (7.4±0.2)×10^−11^ and (7.9±0.2)×10^−11^ m^2^/sec, respectively, without any indication of biphasic behavior. Faster diffusion of BteA32-287 cannot be accounted solely by the truncated N-terminal tail, and the 7% change in Ds (as opposed to the 36% observed for BteA287 strongly indicates that the majority of intermolecular forces causing aggregation involved the missing residues 1–31. For globular structured proteins the relation *D*_s_–(MW)^−⅓^ is widely used, although a more general assumption is *D*_s_–(MW)^−a^ with ⅓≤a≤½[15]. This places the estimated molecular weight of BteA32-287 in the 28–39 kDa range, consistent with the calculated value of 30.8 kDa, further demonstrating that residues 1–31 contribute to the aggregation phenomenon observed for BteA287.

### BteA287 is an elongated monomer in solution with a 2:1 aspect ratio

**Figure 6:**
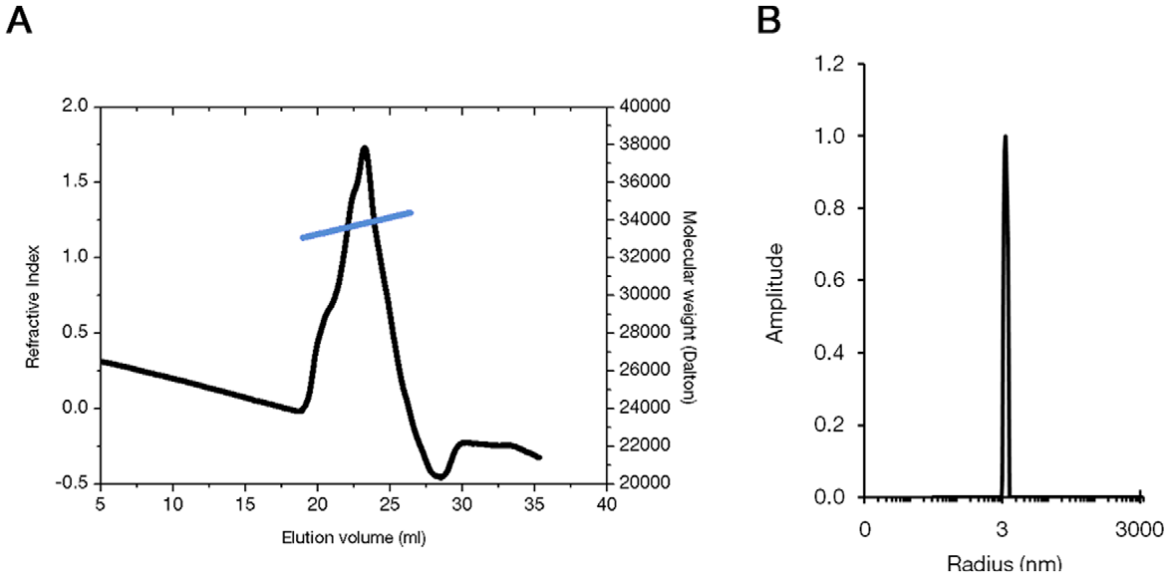
Shape and size distribution of BteA287. (**A**) SEC-MALLS chromatogram shows the elution curve of BteA287 according its refractive index (black line) and the calculated molecular mass of the each eluting protein throughout the peak (colored light blue and scaled on the right-hand axis). (**B**) Dynamic light scattering intensity particle size-distribution of BteA287 at 2.5 mg·ml^−1^.

We utilized size exclusion chromatography coupled to multiangle laser light scattering (SEC-MALLS) and dynamic light scattering (DLS) to further investigate BteA behavior at low protein concentration. SEC-MALLS indicated that BteA287 has an average mass distribution of 33.7±0.4 kDa (Figure 6A, blue line), in perfect agreement with its molecular weight. The apparent multi-peak refractometer reading (Figure 6A, black line) may be accounted for by single amino-acid degradation products which could not be resolved on a Superdex column (Figure 5A) but only on the high resolving Suprema column (see Material and Methods). DLS analysis yielded a unimodal size distribution of 3.5±0.58 nm (Figure 6B). Since a 70 Å diameter is irreconcilable with a monomeric and spherical 34 kDa protein, we deduced that BteA287 adopts an ellipsoidal rather than a spherical shape. Equating the ellipsoid volume with BteA287's theoretical volume (equation 1) we determined the short dimension radius as 1.67 nm long, establishing the protein's aspect ratio as ∼2:1. Taken together, our results suggest that BteA287 behaves in solution as a monomer characterized by an elongated ellipsoid shape with an aspect ratio of 2:1 and a molecular mass of 33.7 kDa.

### SAXS shows BteA287 adopts an elongated bi-pyramidal dumbbell shape

**Figure 7:**
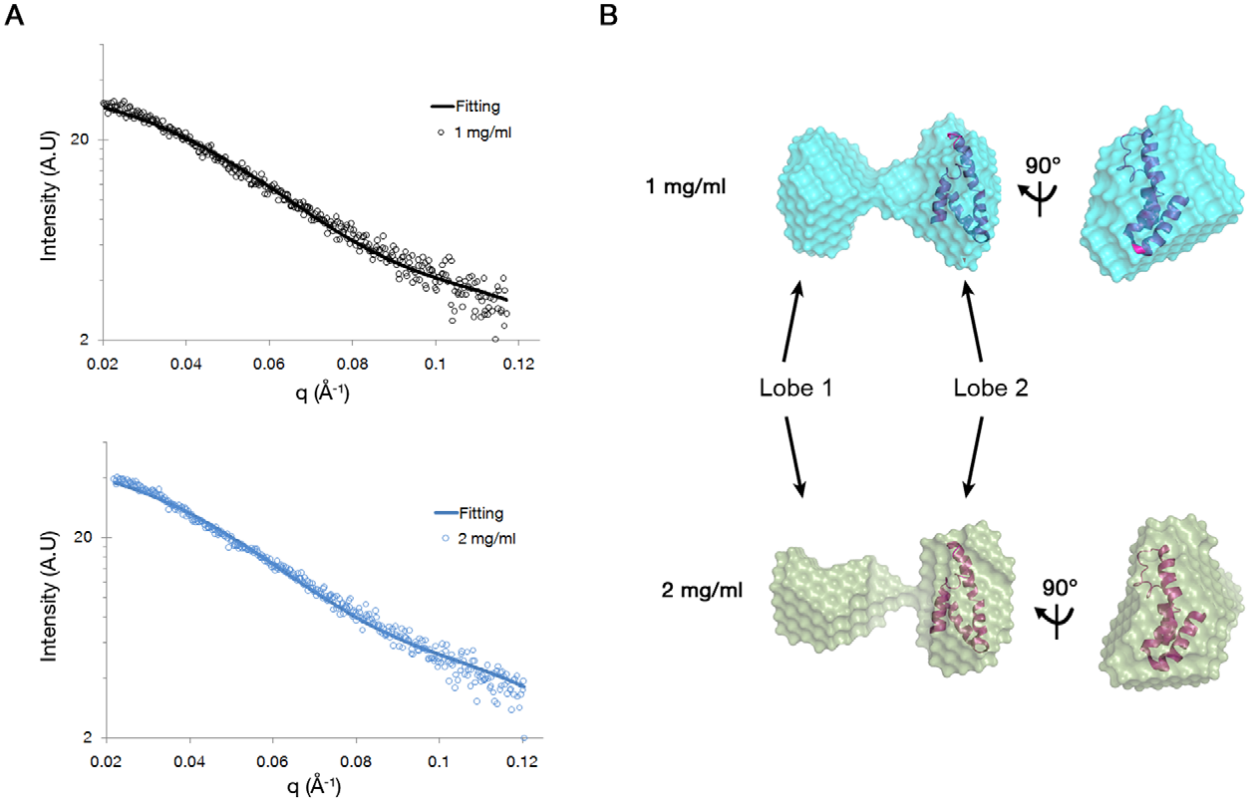
SAXS analysis of the BteA287. (**A**) Experimental data of the BteA287 (dots represent experimental data, and lines show fit) (**B**) Dummy-ball models (DBMs) of mg/ml (30 µM, upper panel) and 2 mg/ml (60 µM, lower panel) fitted onto the core deposited structure of BteA_115–220_ (PDB code 2JPF).

Our observations that BteA287 adopts an ellipsoid shape led us to further investigate its architecture using SAXS experiments. The X-ray scattering data was collected at three different protein concentrations and the scattering plots of 1 and 2 mg/ml protein solutions are shown in figure 7A. At these concentrations the scatterings are quite similar one to another and thus indicate that, in agreement with previous observations, molecular dimensions are independent of concentration over this concentration range. Radii of gyration (Rg) obtained at 1 and 2 mg/ml were 3.04 nm and 3.17 nm, respectively, both within the measured radius of hydration obtained by DLS (3.5±0.58). Notably, at higher concentrations (10 mg/ml) scattering was 2-fold elevated (figure S1) and a larger Rg (4.11 nm) was predicted. Since aggregation is most likely to contribute to these effects, this concentration was deemed inappropriate for further analysis.

To refine our geometric model of BteA287, we generated a dummy-ball model (DBM) from the SAXS data for 1 and 2 mg/ml samples utilizing the DAMMIN software (figure 7B) [34]. Both models displayed the shape of a bi-pyramidal dumb-bell marked by lobe 1 and lobe 2 (figure 7B) and were back fitted on the scattering data with goodness of fit. The solution NMR structure of BteA_115–220_ (PDB code 2JPF) could be superposed on lobe 2 but not on lobe 1 (figure 7B, ribbon model). Despite this bi-lobal shape, we have ruled out the possibility that BteA287 is actually composed of two smaller domains. Further to our earlier trypsinization results, purified BteA287 was exposed to limited trypsin proteolysis followed by SDS-PAGE analysis (figure S2), confirming that BteA287 is composed of a single domain. We have summarized BteA287 oligomerization state and dimensions in table 1 and conclude that BteA287 adopts a bi-pyramidal dumb-bell shape with an average gyration radius of 3.1 nm.

**Table 1:** Summary of biophysical findings for BteA287.

Technique	Observed oligomerization and size estimation^1^
SEC	68 kDa (or an extended 34 kDa)
SEC-MALLS	33.7 kDa
DLS^2^	3.5 nm, 2:1 aspect ratio
Diffusion	30–42 kDa
SE	34.5 kDa
SAXS	3.04¥/3.11 nm¶ (4.11 nm)

1 Values in parentheses relates to high protein concentration (>200 µM).

2 Radius of hydration.

¥ Radius of gyration of 1 mg/ml sample.

¶ Radius of gyration of 2 mg/ml sample.

## Discussion

Previous publications have demonstrated the key importance of the T3SS effector BteA in the cytotoxicity of *B. bronchiseptica* towards cultured cells. It is the only confirmed T3SS effector discovered in any species of the ‘classical’ Bordetella thus far [4]–[6]. To date only a small core region of BteA287 (residues 115–220) has undergone structure determination, and our aim was to unveil the biophysical and biochemical properties of BteA in the context of its oligomeric and structural characteristics. In this study we have focused on the N-terminal domain, a region of 287 residues that encompasses both the chaperone binding site (CBD) and the lipid raft targeting (LRT) region of BteA. We show herein that BteA287 is a highly stable domain rich in alpha helical folds and characterized by an elongated shape composed of bi-pyramidal lobes interconnected in a dumb-bell fashion.

The lack of significant sequence homology to known proteins (other than the lipid raft targeting region), and the ability to cause rapid eukaryotic cell death by unknown means, suggest that BteA possesses a novel structure and possibly unique function(s) within the host cell. Most T3SS effectors have a modular architecture, in which the N-terminal domain contains the secretion signal sequence and a CBD, while the C-terminal domain elicits a relatively subtle function within the host cell [35]. We have found BteA to comprise of N and C-terminal domains (Figures 1 & 2). The N-terminal is characterized by a secretion signal within its first 30 residues followed by overlapping CBD and LRT domains, with the latter required for the localization of BteA within the host cell [4]. Such overlapping functional domains were previously demonstrated for YopO which contains a CBD that overlaps with its periplasmic membrane localization domain (MLD) at its N-terminus such that the chaperone masks the MLD when the effector is located within the bacteria [36]. Similar domain architecture to BteA287 has been found in SipA which has two modular N and C-terminal domains interconnected by an insoluble linker domain [37], [38].

A hallmark of BteA is its ability to form SDS-resistant high molecular weight oligomers, as demonstrated in this study with its recombinant form and previously with the endogenous molecule [4]–[6]. In contrast, we found the truncated BteA287 to be highly soluble with no trace of the SDS-resistant oligomers, suggesting this phenomenon requires the presence of the C-terminal domain (Figure 3). Furthermore, we found BteA287 to be efficiently expressed in *E. coli* with no observable inclusion bodies (data not shown).

In this work we employed a combination of biophysical methods to show BteA287 to be a highly helical peptide, and demonstrate that the N-terminal domain of BteA adopts an elongated shape rather than a spherical one. This was evident from the results of analytical SEC, SEC-MALLS analysis, and SAXS envelope models, all of which were in agreement that BteA287 behaves as a prolate ellipsoid, with an estimated aspect ratio of ∼2:1. We found that BteA287 has a concentration-dependent behavior in which, at higher concentrations (>200 µM), the protein shifts into a monomer-dimer equilibrium with the appearance of a small fraction of aggregates (as seen by the SE experiments). We assume that this concentration-dependent oligomerization has no relevance at the physiological level within the host cells, in which the full length BteA binds to its cognate protein(s) host through this domain.

In the context of the concentration-dependent behavior of BteA287, NMR was utilized as a tool for the initial characterization of BteA287, capable of providing biophysical information even in the absence of a structure and at relatively high protein concentrations. Diffusion spectroscopy, sensitive to the hydrodynamic size of the polypeptide, was applied to BteA287 to identify its translational motions, and thus determine its size. NMR also uncovered the tendency of BteA287 to aggregate at higher concentrations, as revealed by its concentration-dependent diffusion behavior and the concentration effects on the NMR spectrum. Our demonstration that the first 31 residues contribute to the aggregation of BteA287 further supports the notion that one of the T3SS chaperon's roles is to protect the pathogenic bacteria from self effector's toxicity and aggregation events. Similar demonstration was shown for YopO, which its CBD lead to acute aggregation and required the fusion of GST or interaction with its cognate chaperon, SycO [36].

The SAXS envelope models are a first glimpse toward a detailed structure of the BteA N-terminal domain. Although the low resolution envelope models exhibit a bi-lobal shape, our trypsinization experiments clearly establish BteA287 as a single stable domain, in which the existing structure (residue 115–220) must reside in the larger lobe 2. A comparison of this BteA architecture to other T3SS effectors reveals that the bi-pyramidal shape of BteA287 is unique. The N-terminus of SipA, as determined by x-ray crystallography, has a globular shape [38], a characteristic of many T3SS effectors. The N-terminal of ExoS, on the other hand, is characterized by an elongated shape (2:1) though its N-terminal only comprises its GAP function [39]. In both SipA and ExoS the N-terminal domain exhibits a single chaperone-binding function. BteA287, in contrast, is involved in additional role of BteA localization within the host cell. It is possible that this dual role of BteA287 is correlated with its bi-lobal structure established in this investigation, although further studies will be necessary to substantiate this claim.

In summary, we have applied a wide range of biophysical methods to characterize the N-terminal BteA287 domain in solution. We have determined BteA287 to be a monomeric 33.8 kDa protein which assumes an elongated bi-lobal shape with an approximate aspect ratio of 2:1, and this is most probably its functional state in the cell. Its exhibited aggregation tendency, assumed to have no biological role and which is the result of the unstructured first 31 residue of BteA, leads to concentration-dependent oligomerization behavior, which typically manifests itself at concentrations above 6–8 mg/ml (or ∼200 µM). Our biophysical and NMR results lay the foundations for further structural studies of this intriguing *Bordetella* effector.

## Supporting Information

Figure S1 **SAXS experiment of BteA287 at 10 mg/ml.** Experimental data of BteA287 at 10 mg/ml. Inset, Guinier plot (squares) with fitted correlation line (red).
(TIF)

Figure S2 **BteA287 limited proteolysis with Trypsin.** Purified BteA287 was mixed with 1.5 mg/ml of Trypsin at a ratio of 1:5000 and incubated at room temperature for the indicated time points at which the reaction was quenched with equal volume of sample buffer. Samples were resolved on a 17.5% SDS-PAGE and stained with coomassie blue stain.
(TIF)
