# Supplementary figures and images for: Characterization of the N-Terminal Domain of BteA: A Bordetella Type III Secreted Cytotoxic Effector
Source: PLoS One. 2013 Jan 30;8(1):e55650. doi: 10.1371/journal.pone.0055650 (PMC3559503; doi:10.1371/journal.pone.0055650)

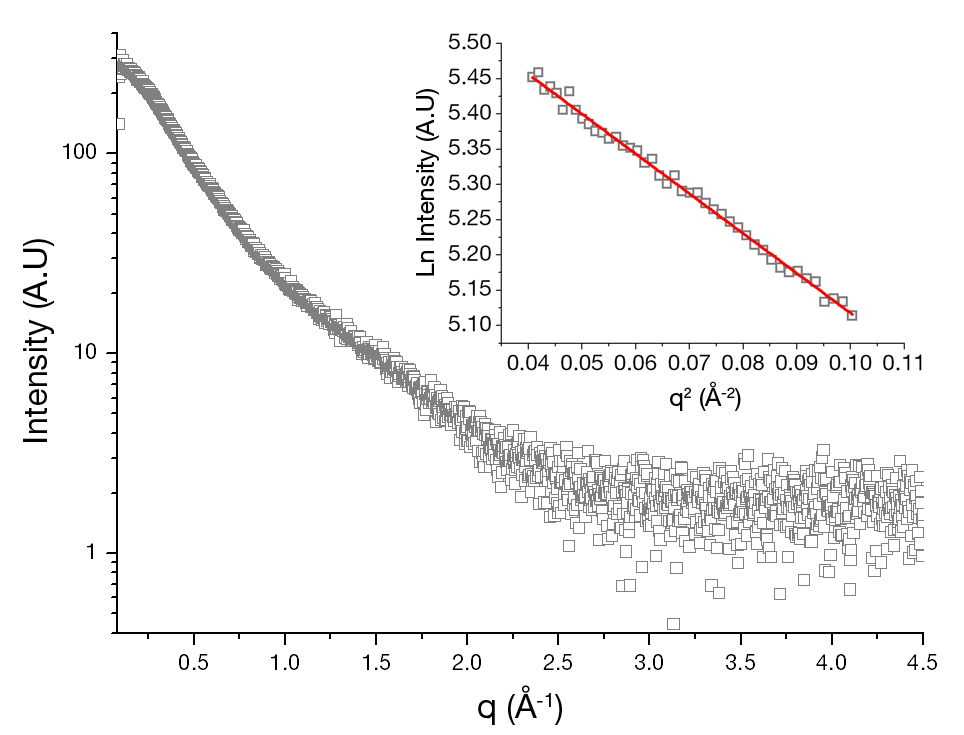

Supplement: Figure S1 — SAXS experiment of BteA287 at 10 mg/ml. Experimental data of BteA287 at 10 mg/ml. Inset, Guinier plot (squares) with fitted correlation line (red). (TIF) [file pone.0055650.s001.tif]

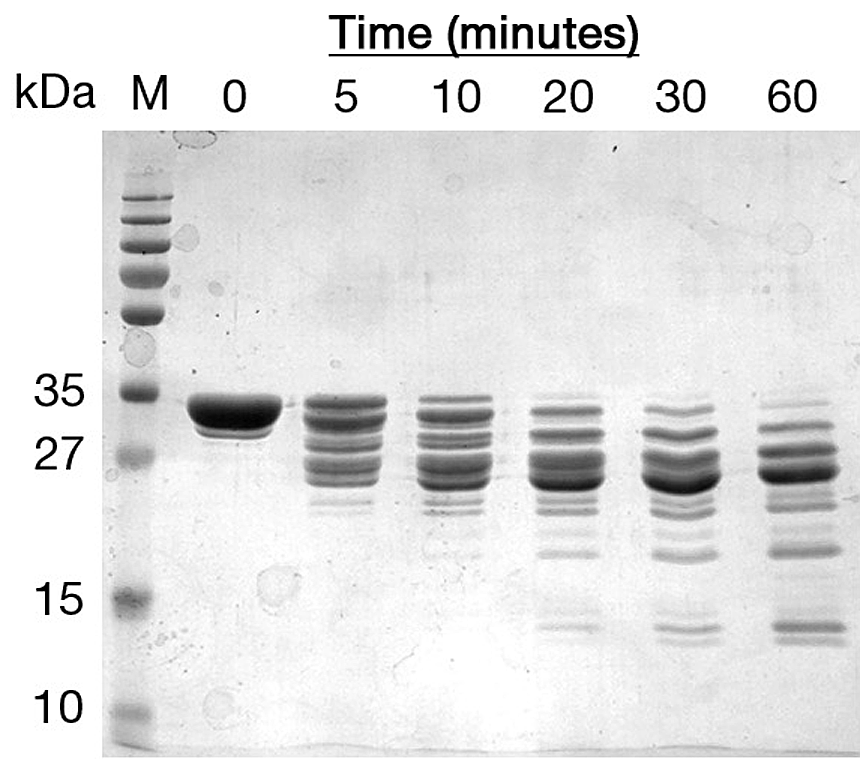

Supplement: Figure S2 — BteA287 limited proteolysis with Trypsin. Purified BteA287 was mixed with 1.5 mg/ml of Trypsin at a ratio of 1:5000 and incubated at room temperature for the indicated time points at which the reaction was quenched with equal volume of sample buffer. Samples were resolved on a 17.5% SDS-PAGE and stained with coomassie blue stain. (TIF) [file pone.0055650.s002.tif]
